# Vascularizing the brain *in vitro*

**DOI:** 10.1016/j.isci.2022.104110

**Published:** 2022-03-17

**Authors:** Abdellah Aazmi, Hongzhao Zhou, Weikang Lv, Mengfei Yu, Xiaobin Xu, Huayong Yang, Yu Shrike Zhang, Liang Ma

**Affiliations:** 1 State Key Laboratory of Fluid Power and Mechatronic Systems, Zhejiang University, Hangzhou 310058, China; 2 School of Mechanical Engineering, Zhejiang University, Hangzhou 310058, China; 3 The Affiliated Stomatologic Hospital, School of Medicine, Zhejiang University, Hangzhou 310003, China; 4 School of Materials Science and Engineering, Tongji University, Shanghai 201804, China; 5 Division of Engineering in Medicine, Brigham and Women’s Hospital, Harvard Medical School, Cambridge, MA 02139, USA

**Keywords:** Biotechnology, Tissue Engineering, Materials science, Biomaterials

## Abstract

The brain is arguably the most fascinating and complex organ in the human body. Recreating the brain *in vitro* is an ambition restricted by our limited understanding of its structure and interacting elements. One of these interacting parts, the brain microvasculature, is distinguished by a highly selective barrier known as the blood-brain barrier (BBB), limiting the transport of substances between the blood and the nervous system. Numerous *in vitro* models have been used to mimic the BBB and constructed by implementing a variety of microfabrication and microfluidic techniques. However, currently available models still cannot accurately imitate the *in vivo* characteristics of BBB. In this article, we review recent BBB models by analyzing each parameter affecting the accuracy of these models. Furthermore, we propose an investigation of the synergy between BBB models and neuronal tissue biofabrication, which results in more advanced models, including neurovascular unit microfluidic models and vascularized brain organoid-based models.

## Graphical abstract

**Figure:**
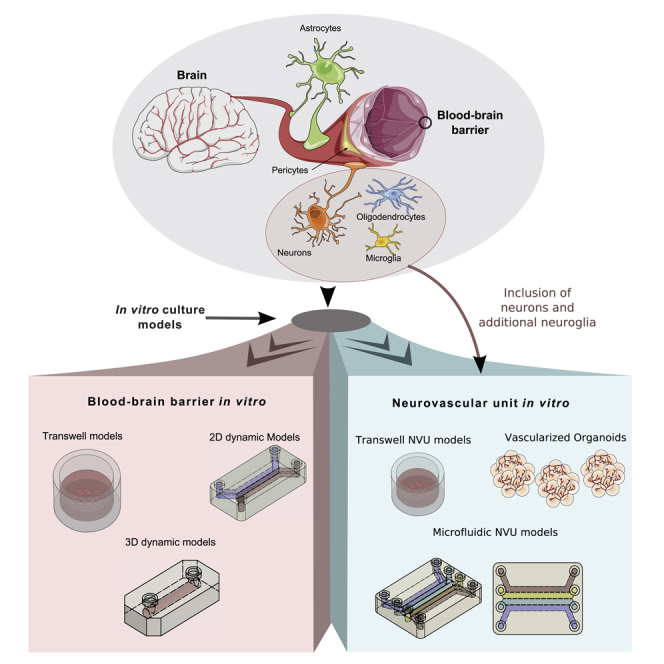

## Introduction

The human brain represents the most intricate organ with its various and numerous functions that remain mysterious. It is connected to the spinal cord to form a central nervous system (CNS) controlling other body parts. Defining the physiological nature of the CNS has been ambiguous until the breathtaking findings of Golgi and Cajal confirmed that the human brain structure is not totally different from other organs (Glickstein, 2006). Indeed, it is a tissue composed of discrete cells and vasculature (Kandel et al., 2000). It had been hardly believed that our thoughts, senses, and actions are the results of neural signals until it was proven a century ago but still not fully elucidated nowadays. To fulfill the scientific need to understand the brain functions, besides *in vivo* systems, *in vitro* models have been of great help to closely investigate different aspects of the brain (Bang et al., 2019; Mofazzal Jahromi et al., 2019; Tang-Schomer et al., 2014). Investigating biological systems for drug discovery and toxicity testing are two of the critical research routes offered by these models(Ma et al., 2020b, 2020c). Both these pathways have a common starting point of approaching the normal or pathological functions of the brain. Therefore, *in vitro* brain models need to be validated first, based on specific parameters characterizing the real organ.

To replicate the CNS *in vitro* is an interdisciplinary field that has drawn much attention during the last decade (Bhalerao et al., 2020; Hopkins et al., 2015; Keep et al., 2021; Lee and Leong, 2020; Neumaier et al., 2021; Teixeira et al., 2020). The neuronal tissue, the vasculature, and the interstitial system are the three main components that make up the brain. Despite their interconnected roles, many existing *in vitro* models address each part separately (Gradisnik et al., 2021; Hanafy et al., 2021; Ma et al., 2020b; Singh et al., 2021; Yang et al., 2021). Therefore, research advances showed to be dispersed, not fully connected, and limited by dissociated parameters set to create the *in vitro* model. Creating vascularized *in vitro* models is now considered as a more accurate approach to recreate different *in vivo* organs and pathologies (Li et al., 2021b; Ma et al., 2020a). Remarkably, the brain vasculature is an important aspect of the brain that has recently attracted increasing attention because of the need to replicate its intricate functions and structure.

**Figure 1:**
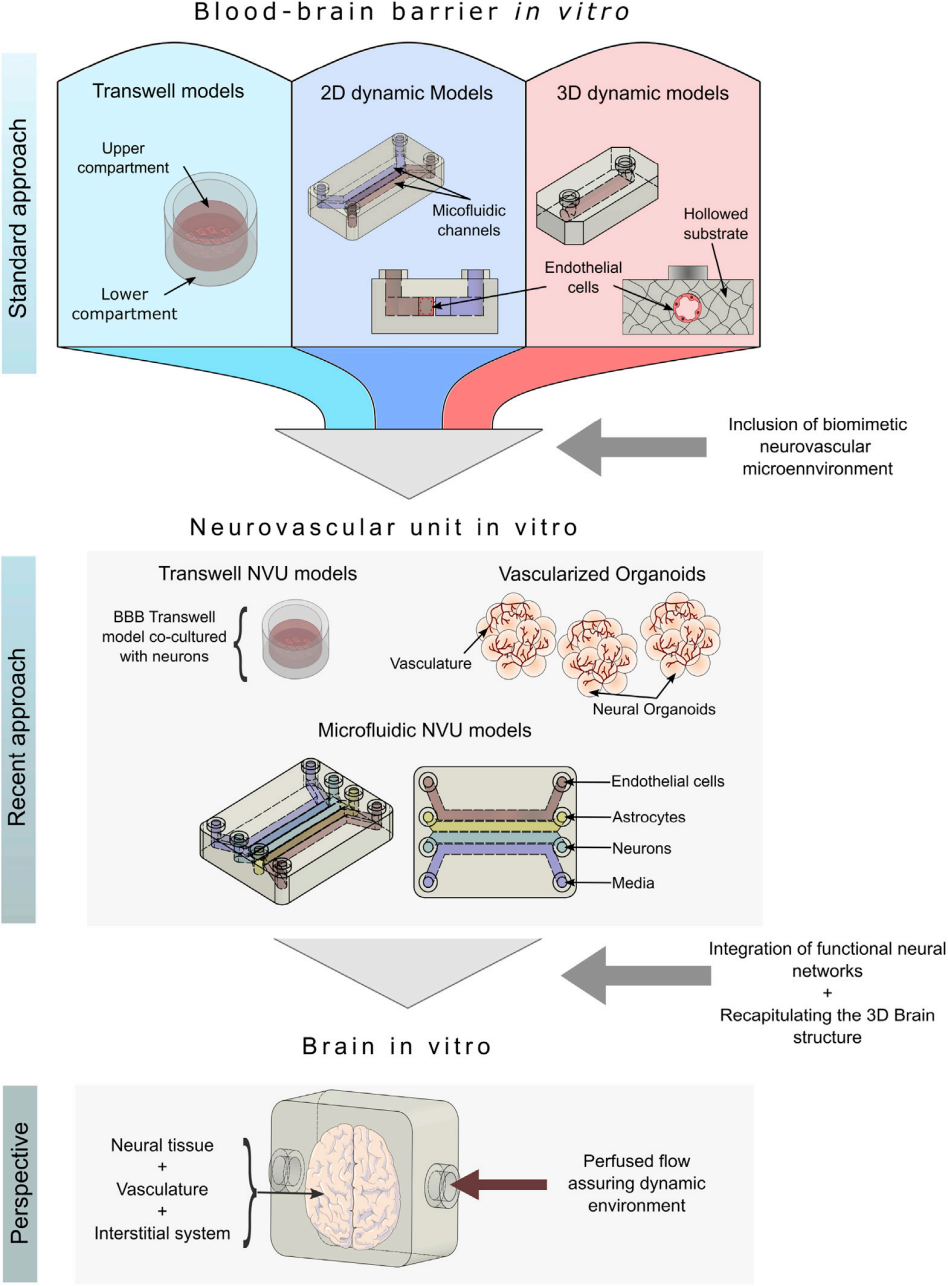
Schematic illustration of the current advances and perspective in BBB *in vitro* models.

In this review, we first describe the structure and the functions of the brain vasculature and then define and analyze different approaches used to mimic this specific vasculature *in vitro*. Finally, we present a synergy closing the gaps between different approaches to attempt a redirection of research works toward engineering more accurate vascularized brain *in vitro* models (Figure 1). The abbreviations used in this paper are listed in Table 1.

**Table 1:** List of abbreviations

Abbreviation	Definition
αSMA	α-smooth muscle actin
μ-PIV	Micro-particle image velocimetry
ABC	ATP cassette binding
AC	Astrocyte
AJ	Adherens junctions
BBB	Blood-brain barrier
BBBoC	BBB-on-a-chip
BM	Basement membrane
BMECs	Brain microvascular endothelial cells
CAR	Coxsackie adenovirus receptor
CD31	Cluster of differentiation 31
CNS	Central nervous system
dECM	Decellularized extracellular matrix
EC	Endothelial cell
ECM	Extracellular matrix
EGM	Endothelial growth medium
ESAM	Endothelial cell-selective adhesion molecule
FITC	Fluorescein isothiocyanate
GCX	Glycocalyx
GFAP	Glial Fibrillary Acidic Protein
hES	Human embryonic stem cell
hiPSC	Human induced pluripotent stem cell
hPSC	Human pluripotent stem cell
HUVEC	Human umbilical vein endothelial cell
INPM	Indoor nanoscale particulate matter
IL-1	Interleukin-1
iPSC	Induced pluripotent stem cell
ISS	Interstitial system
MACS	Magnetic-activated cell sorting
MCP-1/2	Monocyte chemoattractant protein-1/2
MEM	Minimum essential medium
MSC	Mesenchymal stem cell
NG2	Neural/glial antigen 2
NOD-SCID	Non-obese diabetic/severe combined immunodeficiency
NPCs	Neural progenitor cells
NSC	Neural stem cell
NVU	Neurovascular unit
OOCs	Organs-on-chips
P-gp	P-glycoprotein
PC	Pericyte
PDGF	Platelet-derived growth factors
PDMS	Polydimethylsiloxane
PECAM-1	Platelet endothelial cell adhesion molecule-1
PEG-RGD	Poly-(ethyleneglycol) arginine-glycine-aspartic acid
PET	Polyethylene terephthalate
PS	Polystyrene
SHH	Sonic Hedgehog
SiN	Silicon nitride
TAMPS	TJ-associated MARVEL proteins
TEER	Transepithelial/transendothelial electrical resistance
TGF- β	transforming growth factor β
TJ	Tight-junction
TNF-α	Tumor necrosis factr- α
VEGF	Vascular endothelial growth factor
VFP	Viscous finger patterning
ZO	Zonula occludens
μBBB	Microfluidic BBB model

## The BBB

**Figure 2:**
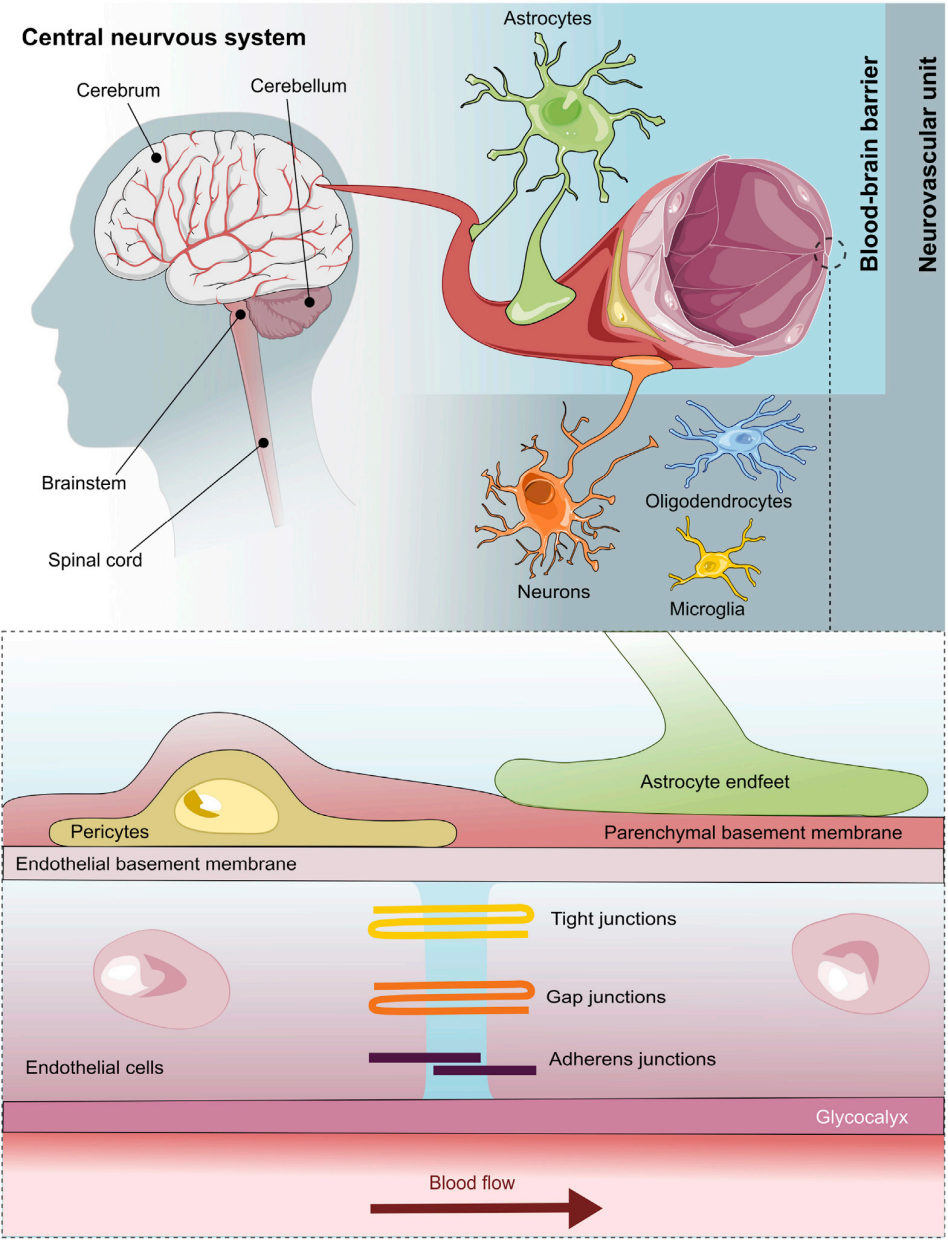
Illustration of the structure of CNS, NVU and the BBB.

The presence of a tight and selective barrier protecting the brain from neurotoxic compounds and microorganisms is a distinguishing feature of the brain vascular system. However, it is essential to note that this characteristic does not apply to all the brain vasculature; in fact, the circumventricular organs, which are midline structures in the walls of the third and fourth ventricles, are distinguished by the existence of fenestrated capillaries and rather extensive perivascular gaps (Oldfield and McKinley, 2015). The CNS regulates this barrier, termed the blood-brain barrier (BBB), to maintain and regulate the brain microenvironment (Figure 2). The BBB is formed through a complex process induced by multiple growth factors and signaling pathways, including vascular endothelial growth factor, platelet-derived growth factors (PDGF-B and PDGFR-β), canonical Wnt signals, and Sonic Hedgehog signals (SHH); detailed aspects can be found elsewhere (Bauer et al., 2014; Daneman and Prat, 2015; Engelhardt and Liebner, 2014; Liebner and Plate, 2010; Sweeney et al., 2019). This barrier is composed of brain endothelial cells, pericytes (PC), basement membrane (BM), and astrocyte (AC) endfeet. The BBB is surrounded by several types of neuronal cells, which also play an essential role in its formation and maintenance, including neurons, microglia, oligodendrocytes, and neural/glial antigen 2 (NG2)-expressing cells. These cells, along with the BBB, form the neurovascular unit (NVU), which is primarily responsible for the BBB plasticity allowing it to be an active cellular structure.

### Cells forming the BBB

#### Endothelial cells

The brain endothelial cells (ECs) have specialized functions and characteristics, making them more suited to assure the homeostasis of such a delicate organ as the brain. Unlike peripheral blood vessels, the BBB ECs are structurally characterized by their lack of fenestrae, low pinocytosis, flattened shape, and a high number of mitochondria (Castro Dias et al., 2019; Kadry et al., 2020). In addition, ECs are continuously exposed to several mechanical cues induced by hemodynamic forces: shear stress and circumferential stretch (Dessalles et al., 2021). The pathways across the BBB are minimal compared to other endothelial barriers (Uwe and Hartwig, 2008). The existence of a distinct network of receptors, transporters, non-selective drug export pumps, and specific paracellular junctions that are more similar to those seen in epithelial barriers restrict paracellular and transcellular diffusion across the BBB (Uwe and Hartwig, 2008).

#### Perivascular cells

The BBB’s perivascular cells generally include only PCs and neuroglia, whereas 10% of the brain vasculature have arterial and venous smooth muscle. PCs are the closest cells to the BBB ECs, which are separated by a secreted extracellular matrix (ECM). Their primary function is to maintain and regulate the growth of the BBB and control blood flow in response to neuronal activities (Brown et al., 2019a). In addition, they have played an essential role as immune-mediators in the CNS (Rustenhoven et al., 2017). They also have the multipotential ability to differentiate into vascular and neural lineage cells, including microglia, neural progenitors, vascular cells, and angioblasts (Nakagomi et al., 2015).

Compared to other non-neural cells, ACs are a predominant cell type performing a wide range of functions that significantly impacts the brain (Miller, 2018). The prominent roles of ACs include synaptic regulation, ion homeostasis, water homeostasis, maintenance of cellular pH, the providence of energetic metabolites required by neurons, maintenance of the BBB, and finally, forming a system of tunnels in the glymphatic system to remove or distribute some specific molecules (Matias et al., 2019). Based on their morphologies, ACs can be classified into two categories: The protoplasmic ACs, which are located in the gray matter, and the fibrous ACs, which are found in the white matter (Miller, 2018). Recent research has discovered significant AC heterogeneity based on their differential expressions of GLT-11/SLC1A2, intrinsic membrane properties, neuronal maturation and synaptogenesis, and Ca^2+^ signaling (Hasel et al., 2021; John Lin et al., 2017; Tabata, 2015){John Lin, 2017 #291}.

### Junctional molecules in the BBB

The cell-cell connection in the BBB ECs is assured by junctional molecules, which are tight junctions (TJs), gap junctions, and adherens junctions (AJs) (Sweeney et al., 2019). TJs are composed of transmembrane proteins which limit the paracellular diffusion of ions and solutes; cytoplasmic scaffolding proteins; cytoskeletal polymers; membrane lipids (Citi, 2019). The transmembrane proteins are claudins; immunoglobulin-like cell-adhesion molecules include JAM-A, coxsackie adenovirus receptor (CAR), EC-selective adhesion molecule (ESAM), and angulins; TJ-associated MARVEL proteins (TAMPS) such as occludin, tricellulin, and MarvelD3 (Castro Dias et al., 2019; Citi, 2019; Greene et al., 2019). The transmembrane proteins are connected to cytoskeletal polymers actin filaments and microtubules by interacting with cytoplasmic scaffolding proteins such zonula occludens (ZO) proteins (ZO-1, ZO-2, ZO-3) and the membrane-associated guanylate kinase inverted proteins (MAGI-1 and MAGI-3) (Arvanitis et al., 2020). Moving toward the abluminal side of the BBB, gap junctions connexin (CX)-37, CX40, and CX43, as well as AJ VE-cadherin, and platelet endothelial cell adhesion molecule-1 (PECAM-1) complement the TJs by maintaining the BBB integrity and ensuring the intracellular communication (Arvanitis et al., 2020). The involvement of membrane lipids in TJ is unclear; however, it is a promising subject for future investigation(Shah et al., 2018; Shigetomi et al., 2018).

### Substrates covering the BBB: the BM and glycocalyx

From the abluminal side, the BBB is covered by an ECM of two layers to form the vascular BMs. The two layers of the BM are the endothelial BM secreted by ECs and the parenchymal BM secreted by ACs, which are separated by PCs. The vascular BM has a thickness of 20–200 nm, which is composed of four major glycoprotein families, including collagen type IV isoforms, laminins, nidogen, and heparan sulfate proteoglycans (Thomsen et al., 2017; Xu et al., 2019). The main difference between parenchymal and endothelial BM resides in their laminin compositions. The parenchymal BM is characterized by the combination of laminin *α*4 and *α*5 chains with laminin *β*1 and *γ*1 chains to form laminin isoforms 411 and 511, respectively (Engelhardt and Sorokin, 2009). The endothelial BM, on the other hand, has laminins isoforms 111 and 211, which are made up of laminin *α*1 and *α*2 chains combined with laminin *β*1 and *γ*2 chains (Engelhardt and Sorokin, 2009). Because of the BM’s thin thickness and strong attachment to the surrounding cells, its biomechanical properties such as stiffness and permeability are yet uncertain, though the Young’s modulus is reported to a range from a few kPa to a few MPa (Candiello et al., 2007; Li et al., 2021a; Miller, 2017).

The luminal side of the BBB is covered by a thin and dense layer of a villiform substance secreted and synthesized by ECs, which is termed glycocalyx (GCX) (Jin et al., 2021; Kutuzov et al., 2018). It is composed of proteoglycan polymers and glycosaminoglycan chains, including heparan sulfate, chondroitin sulfate, and hyaluronan (Jin et al., 2021). Recent studies proved the importance of the presence of this substrate since it has a vital role in preventing coagulation and regulating the BBB permeability and the occurrence and development of inflammation (Ando et al., 2018; Santa-Maria et al., 2019; Zhu et al., 2018, 2021a).

## *In vitro* models of the BBB

### BBB transwell models

The BBB Transwell models generally involve the formation of a thin layer of brain ECs using a permeable membrane submerged in an *in vivo*-like microenvironment. The Transwell models are composed of two compartments, separated by a porous membrane, including the upper compartment that usually contains brain ECs, and the lower compartment that might include glial cells. The porous membranes are characterized by their insert growth area, pore sizes ranging from 0.4 to 3 μm, pore density, and cytocompatibility. These four parameters directly affect the cell morphologies and the TJ formation (Chung et al., 2018; Ferro et al., 2020; Vigh et al., 2021).

**Figure 3:**
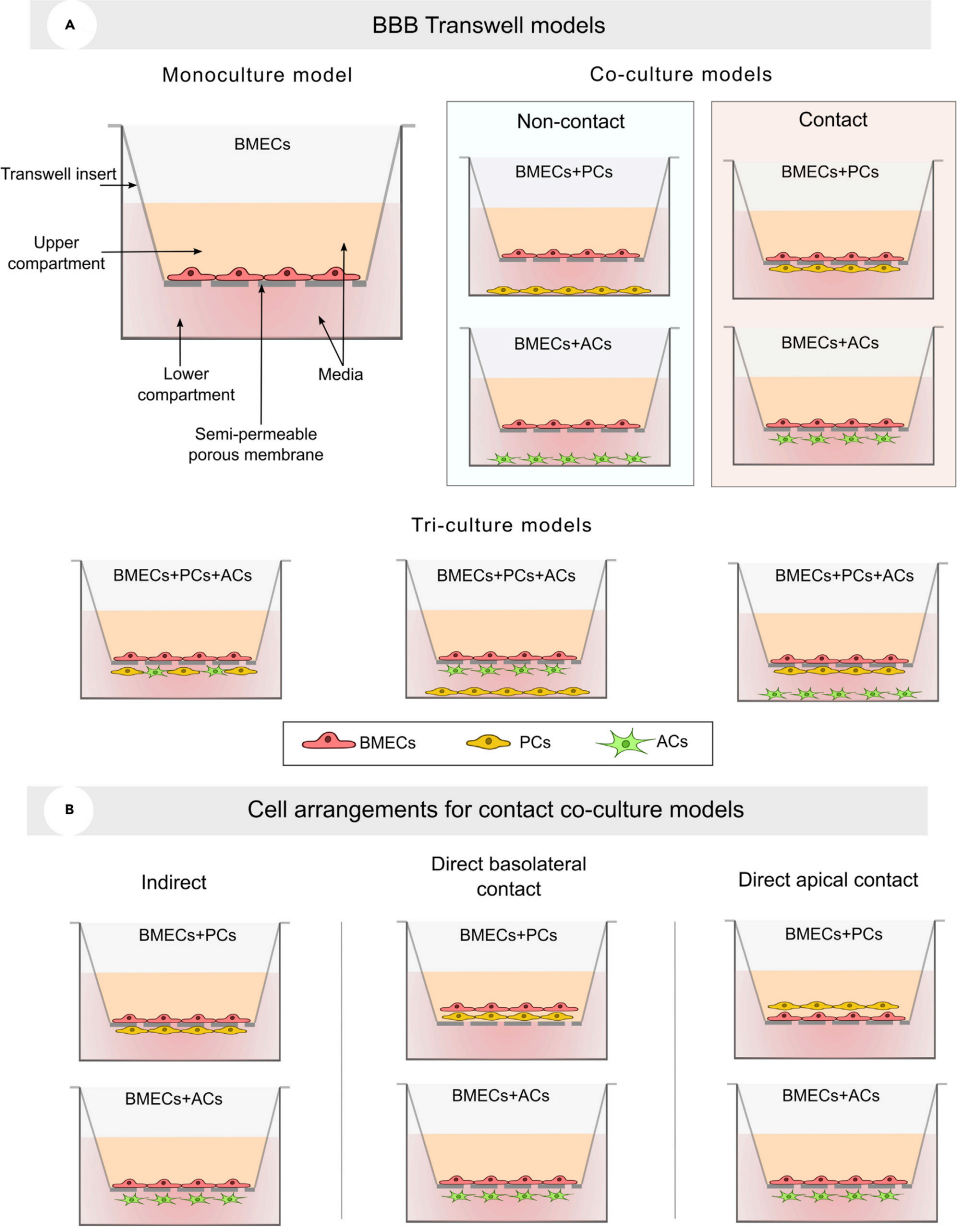
Schematic illustration of Transwell models (A) Monoculture, co-culture and tri-culture BBB Transwell models. (B) Cell arrangements for contact co-culture models.

The Transwell models are considered simple to replicate, inexpensive, and easily assessable. However, their failure to mimic real dynamic conditions and ensure cell-cell contact, which is limited by the porous membrane, is a significant flaw that drives researchers to use dynamic culture models regardless of their complexity. Using a Transwell culture model, researchers have been able to approach the BBB in two different ways, depending on the model’s intended complexity (Figure 3A). The monoculture and co-culture procedures are the two most commonly utilized cell culture strategies.

#### Transwell monoculture models

The monoculture model, in which only the brain ECs are presented on the permeable membrane, is the simplest model with the least accuracy. The Transwell membrane, at its role, mimics the brain vascular lumen; meanwhile, the narrowing compartments are used to maintain and assess the formed endothelial barrier overlooking the critical role of parenchymal cells in developing the TJ results in a significantly low transepithelial/transendothelial electrical resistance (TEER) value. Indeed, the TEER measurements for monoculture are reported to be less than other culture models. The barrier tightness can be improved by adding glucocorticoids dexamethasone and hydrocortisone, which increase most TJ protein expressions, namely occludin, claudin-1, claudin-12, ZO-1, and VE-cadherin (Hoheisel et al., 1998; Neumaier et al., 2021; Schrot et al., 2005). The increase of these proteins correlates with the increase of TEER and reduction of permeability. The monolayer model’s simplicity, on the other hand, makes it ideal for studying transport dynamics and cell signaling pathways. However, the findings of this strategy should be validated using an *in vivo* model or a more advanced *in vitro* model.

#### Transwell co-culture models

For better recreation of the BBB *in vitro*, brain microvascular ECs (BMECs) can be co-cultured with additional cell types, such as ACs or PCs. Early *in vitro* studies proved the critical role of ACs and PCs in the TJ formation (Abbott et al., 2006; Armulik et al., 2010; Janzer and Raff, 1987). However, the role of ACs in maintaining barrier integrity was called into question by demonstrating the inaccuracy and lack of evidence in previous research studies (Kubotera et al., 2019; Schreiner et al., 2015). Recent *in vivo* investigations based on comparably accurate BBB permeability assessment methods, contrarily, found that AC ablation disrupted the barrier, which was not replenished by proliferation or any other process extension near the ACs (Ezan et al., 2012; Heithoff et al., 2021). On the other hand, PCs can modify paracellular permeability in cerebral ECs by modulating genes such as occludin and transforming growth factor β (TGF-*β*). As a result, ACs, like PCs, cannot be left out in the creation of a functional BBB *in vitro*. PCs, on the other hand, can significantly improve BBB tightness as compared to ACs (Nakagawa et al., 2007).

The non-contact co-culture and the contact co-culture are two distinct conditions in which the Transwell-based BBB models can be structured. For the co-culture of BMECs and ACs, BMECs are cultivated in the top layer of the Transwell facing the upper chamber whereas ACs can either be in the bottom of the lower chamber (non-contact approach) or on the opposite side of the Transwell membrane, which will allow crosstalk with BMECs (contact approach). The non-contact approach is more doable, yet the contact approach could exhibit an increase in the expression of the gene encoding the TJ proteins claudin-5, occluding, and ZO-1, which then tightens the barrier (Idris et al., 2018). Therefore, the contact co-culture of BMECs and ACs is preferred to increase the BBB integrity. BMECs can also be co-cultured with PCs; however, only the contact approach might be valid at this time. Assuring the contact between PCs and BMECs is not a sufficient condition, but also the direction of the contact must be taken into consideration. Several studies have overlooked this characteristic, leading to the incorrect assumption that PCs play a minor role in the BBB development.

BMECs can also be co-cultured with PCs to mimic the BBB. The contact and non-contact approaches lead to dissimilar TEER and permeability values. However, the placement of PCs and BMECs is not the only structural parameter that affects the BBB functions, but also the arrangement of the contact must be taken into consideration. Kurmann et al. analyzed the TEER and permeability of three possible arrangements of the PCs and BMECs co-culture models (Figure 3B) (Kurmann et al., 2021). As a result, the indirect contact co-culture of PCs and BMECs proved to have a better chance of accurately assessing the influence of PCs on the BBB functions and integrity (Kurmann et al., 2021). Therefore, current contact and non-contact approaches have both been lacking in the accuracy of mimicking the *in vivo* BBB structure. It is mainly due to the limited potential of current biofabrication techniques used in the Transwell models that do not allow precise cell positioning and orientation control.

#### Transwell tri-culture

Because of their critical functions in the BBB integrity, researchers oftentimes further culture both PCs and ACs with brain ECs. ACs and PCs are involved in synergistic inductive functions, such as the necessity of implying ACs to form a more accurate association of PCs and brain ECs. Brain ECs are cultured in the upper side of the Transwell membrane; PCs are cultured on the lower side of the membrane, and ACs in the bottom of the lower compartment. At the *in vitro* level arranging and positioning the cells is not evident. Several known and unknown parameters influence cellular behaviors. Therefore, gathering all cell types present on the *in vivo* BBB is not sufficient to obtain an accurate BBB *in vitro* model. For example, an early study discovered that co-culturing BMECs and ACs result in a TJ with a higher TEER and lower permeability than a tri-culture (Hatherell et al., 2011). This conclusion should be reevaluated and not accepted based on earlier research demonstrating the critical role of PCs. Several factors, such as incorrect cell orientation or other parameters constrained by the Transwell technology itself, could have resulted in these inconsistent results. The thickness of the Transwell membrane, which is limited to 10 μm, is one of the elements that influence the culture of PCs more specifically, as ACs have shown to maintain interactions with BMECs even when they are cultivated comparably away from the membrane. However, after demonstrating its accuracy compared to other approaches, new studies are gravitating more toward the tri-culture approach. Finally, Transwell models cannot be considered entirely reliable due to their lack of dynamic conditions.

### Dynamic models

The major goal of dynamic models is to replicate the realistic dynamic microenvironments of the BBB and hence overcome the limitations of the Transwell models. Generally, the dynamic BBB models are constituted of at least two regions. Monocultured BMECs are cultured in the first region whereas glial cells are usually found in the other regions. The hemodynamic flow generates the shear stress on the luminal surface of the BBB. The benefits of this shear stress on the BBB phenotype are now evident and experimentally proven. Indeed, the shear-induced frictional force causes ECs to stretch and, more critically, activates many mechanotransduction pathways (Ando and Yamamoto, 2009; Takeshita et al., 2014). Additionally, in a fascinating study, Figarol et al. provided additional evidence of the importance of shear stress in, more specifically, *in vitro* BBB tri-culture models by comparing gene expression of TJs under dynamic and static conditions (Figarol et al., 2020). As a result, applying shear stress on the BBB improves its tightness (Santaguida et al., 2006). Experimentally the flow shear stress (τ) can be approximated using flow velocity (Q), the dynamic viscosity (μ), and the radius (R) for cylindrical channels in the following equation (Son, 2007):

(Equation 1) $$\tau =6\mu Q/R^{3}$$

For rectangular cross-section channels with a width (W) and height (H), Equation (2) is used instead:

(Equation 2) $$\tau =\frac{6\mu Q}{WH^{2}}\text{∗}\left(1+\frac{\text{H}}{\text{W}}\right){\text{∗f}}^{\prime }\left(H/W\right)$$

where f' (x) is a correction factor used to accurately estimate the shear stress, which mainly depends on the design of the channels (Hartnett and Kostic, 1989).

Defining the shear stress in the *in vitro* models is not evident and needs repetitive experiments. Of interest, a research team was able to predict the shear stress in a BBB microfluidic model with 2.7% error by using Navier-Stokes equations for laminar flows as the governing equations and finite difference methods to approximate the solution (Jeong et al., 2021). The findings of this work provide a valuable tool for optimizing the microfluidic BBB models and determining an *in vivo*-like shear stress while reducing experiment time and repetitive labor. Besides the shear stress, the implementation of a microfluidic flow allows precise control of the chemical microenvironments. Herein, the control of the liquid or gaseous microenvironment can affect many *in vitro* mechanisms such as cell proliferation, cell growth, endothelial migration, and cellular differentiation (Kuzmic et al., 2019; Wu et al., 2018).

Currently, three main categories of microfluidic BBB models exist, including 2D and 3D models. The number of dimensions for 2D and 3D models corresponds to the number of spatial dimensions that will be used during the biofabrication process to approach the *in vivo* BBB.

#### 2D μBBB models

All the dynamic culture models that take the form of a single planar layer or stratified planar thin layers of cells are classified as 2D models. Herein, the only two structural differences between a Transwell model and a 2D microfluidic model are the presence of a dynamic flow and the type of membrane separating the cells, which we will discuss later in this section.

**Figure 4:**
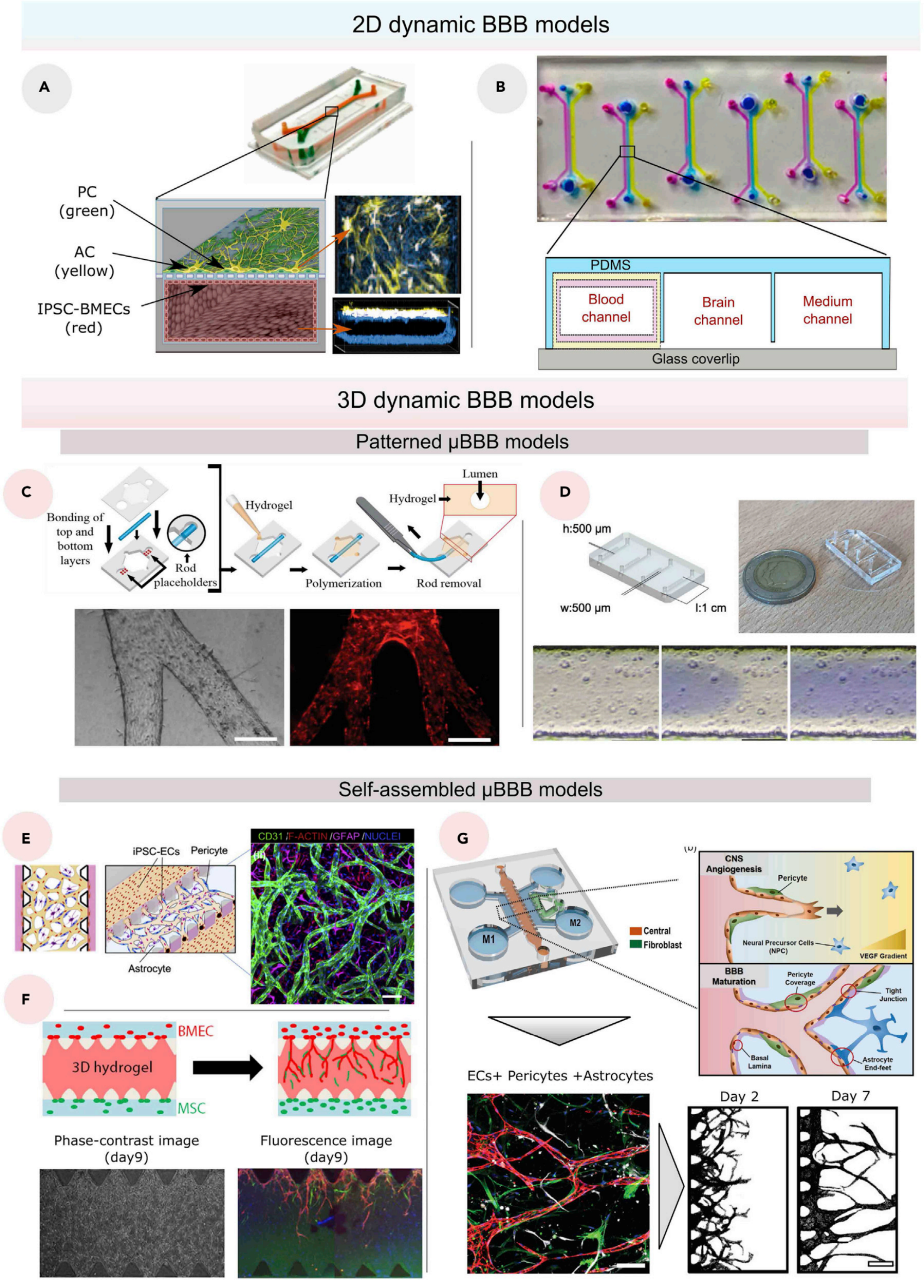
Dynamic BBB models (A) Photograph (top), schematic illustration (bottom left), and immunofluorescence micrographs (bottom right) of a 2D BBB model (Park et al., 2019). (B) Top view (top) and cross section (bottom) of 2D BBB chip composed three channels (Peng et al., 2020). (C) Biofabrication process of a hollowed structure using a rod (top), and bright-field image of bibranched lumen and multibranched (tertiary branches) with phalloidin staining (bottom), with a scale bar of 150 μm (Jimenez-Torres et al., 2016). (D) Images of a hollowed BBB model (top), and time-lapse of viscous finger patterning images showing PBS with blue food dye traveling through collagen solution (bottom), with a scale bar of 200 μm (deGraaf et al., 2019). (E) Schematic illustration (left) and confocal image (right) of a vasculogenesis-based BBB model, with a scale bar of 100 μm (Campisi et al., 2018). (F) Schematic illustration, phase-contrast and fluorescence images of angiogenesis-based BBB model using fibrin-Matrigel mixed gel (Uwamori et al., 2017). (G) Schematic illustration of a BBB angiogenesis-based BBB model sprouting towards a higher gradient of VEGF where the red channel contains the BBB cells (ECs, ACs and PCs), the green channel contains the fibroblasts and the blue channel contains the media (top), confocal image including BMEC (anti-cluster of differentiation 31(CD31), red), ACs (anti-Glial fibrillary acidic protein (GFAP), white), pericytes (anti-α-smooth muscle actin (αSMA), green) and nuclei which were stained with Hoechst 33,342 (blue) (bottom left; with a scale bar of 100 μm), and masked images (bottom right; with a scale bar of 200 μm) of the model (Lee et al., 2020).

The majority of 2D BBB-on-a-chip (BBBoC), also referred to as 2D μBBB models, are based on a previously described protocol by Huh et al. to fabricate an efficient organ-on-a-chip device using a polydimethylsiloxane (PDMS) structure (Huh et al., 2013). The simplicity of the 2D μBBB compared to 3D μBBB can be regarded as a structural disadvantage that can affect the tightness of the barrier. However, several recent techniques have been shown to improve the efficiency of the 2D models considerably. For instance, Park et al. used developmentally inspired differentiation protocols which we have shown previously, to enhance and accelerate the differentiation and expansion of ECs (Han et al., 2010; Kusuma et al., 2014). As a result, the μBBB containing human induced pluripotent stem cell-derived BMECs (hiPSC-derived BMECs) tri-cultured with ACs and PCs, provided a considerably high TEER value (>25,000 Ω) as well as high levels of expressions of TJ and efflux proteins with long-term (up to 2 weeks) functional stability (Figure 4A) (Park et al., 2019).

From another perspective, improving the visualization of 2D models is also a crucial key element that helps assessing the BBB model. Therefore, Peng et al. proposed a stable and homogenously distributed copolymer coating covalently attached to the PDMS structure (Figure 4B) (Peng et al., 2020). The device proved high-throughput screening potential by facilitating the evaluation of paracellular and transcellular pathways. All the 2D culture model parameters have been targeted to improve the accuracy and integrity of the μBBB, yet the planar layers represent a significant limitation since they cannot replicate the ubiquitous tubular structure.

#### 3D μBBB models

The structural limitation of the 2D models has pushed scientists to utilize sophisticated microfabrication and biofabrication methods to recreate 3D curved or tubular BBB surfaces. There are two basic methods in 3D-cultured, patterned, and self-assembled μBBB models, which are distinguished by the biofabrication techniques and substrates used.

##### Patterned μBBB models

To reproduce hollowed structures *in vitro*, a variety of microfabrication techniques have been used. The needle modeling approach was first to be employed (Chrobak et al., 2006). It is based on the patterning of an ECM using a small diameter needle as a mold (Herland et al., 2016; Partyka et al., 2017). For example, after the needle has been inserted into the housing of the microfluidic device, liquid collagen can be introduced to coat the needle (Chrobak et al., 2006). The collagen matrix is then polymerized, allowing us to remove the needle and create a hollowed structure that will support the seeded cells. This technique is reported to be simple to reproduce; however, it can only make one straight hollow cylinder at a time and causes damage to the structure when removing the template. Therefore, Jimenez et al. developed a more sophisticated needle patterning method termed LumeNEXT using a PDMS rod instead of a needle (Figure 4C) (Jimenez-Torres et al., 2016). The PDMS rod can be fabricated either by using a hypodermic needle or polystyrene (PS) molds. The rest of the process remains the same, which means the PDMS rod will be inserted in a housing structure that needs to be filled with an unpolymerized ECM, and then the rod will be removed after the polymerization of the ECM. Unlike the standard needle patterning technique, LumeNEXT is characterized by the tunability of the PDMS rod, which allows the creation of hollow channels with different cross-sectional diameters. LumeNEXT is still not yet applied to the biofabrication of *in vitro* BBB models; herein, its applicability in the culture BMECs still needs to be experimentally validated. After all, manual patterning techniques are not ideal for large-scale applications and demand intensive labor costs.

Another microfabrication technique known as viscous finger patterning (VFP) is based on the use of the gradient of viscosities between two fluids to create hollowed structures (Bischel et al., 2012, 2013). A finger-like shape, termed the Saffman-Taylor finger, is created after the injection of less viscous fluid in another more viscous fluid which is usually the collagen type I hydrogel. In a recent study, deGraaf et al. considered robustness and reproducibility as major characteristics to improve the VFP technique (Figure 4D) (deGraaf et al., 2019). As a result, the research team has successfully optimized the technique by using a combination between the passive pumping and extending the entry length of the injection to create a uniform diameter. Furthermore, whereas the optimized VFP technique is relatively straightforward to scale and more advantageous than the standard VFP technique, it is still limited in creating hollow channels with various cross-sectional diameters and ubiquitous vascular networks. Overall, 3D patterned BBB models provide customizable characteristics for creating hollowed structures, but they are still more difficult to replicate than 2D models and require significantly more time and cost.

##### Self-assembled μBBB models

Current 3D-patterned μBBB models present acceptable structural resemblance to the *in vivo* BBB compared to the 2D models. However, 3D patterning techniques still cannot accurately mimic the complex *in vivo* brain vascular network. Self-assembly techniques, which have previously been utilized to generate normal vascular networks, have the ability to offer a convenient alternative (Kim et al., 2013). As the first attempt to construct BBB using vasculogenesis, Bang et al. co-cultured human umbilical vein ECs (HUVECs) and rat cortical neurons using endothelial growth medium (EGM) in a microfluidic platform (Bang et al., 2017). When tested with 20-kDa and 70-kDa fluorescein isothiocyanate (FITC)-dextran, the model had low permeability coefficients that were consistent with the results *in vivo* (Yuan et al., 2009). Yet, the culture model suffered from two major inconveniences, including its use of umbilical cord ECs rather than brain-derived ECs, and non-employment of PCs whereas they have shown their essential role in the formation of the BBB in other studies (Abbott et al., 2010; Cameron et al., 2021; Wilhelm and Krizbai, 2014).

To address these limitations, Campisi et al. used a similar microfluidic platform, but instead of HUVECs, they used induced pluripotent stem cells (iPSC)-derived ECs co-cultured with PCs and ACs (Figure 4E) (Campisi et al., 2018). Therefore, the vasculogenesis-based BBB model offers a higher BBB integrity, yet it still lacks a parameter related to *in vivo* development of the BBB. The vasculogenesis process happens only during the embryogenesis from the mesoderm to form the perineural vascular plexus via the differentiation of endothelial precursor cells (Liebner et al., 2011). The CNS’s vasculature is mostly formed by angiogenesis, as it is formed from the perineural vascular plexus to infiltrate the embryonic neuroectoderm (Engelhardt, 2003). As a result, despite the progress in vasculogenesis-based models, this strategy cannot be regarded as physiologically relevant since the BBB formation *in vivo* is heavily dependent on CNS angiogenesis (Chico and Kugler, 2021; Umans et al., 2017).

In a recent study, Uwamori et al. have successfully achieved a 3D BBB model based on angiogenesis and neurogenesis approaches by co-culturing neural stem cells (NSCs), BMECs, and human mesenchymal stem cells (MSCs) under an optimized gel condition (Figure 4F) (Uwamori et al., 2017). Unfortunately, the BBB functionalities were not evaluated in this model. Interestingly, Lee et al. were able to recreate a very similar but more efficient culture model without relying on the neurogenesis approach (Figure 4G) (Lee et al., 2020). They investigated the permeability, the functional efflux transporter system, and the vascular morphology to analyze the efficiency model. As a consequence, not only did the method provide an accurate culture model, but it also validated the advantage that the tri-culture of ACs, PCs, and ECs has over other co-culture and monoculture models. Finally, all self-assembled BBB models have a high likelihood of closely resembling the actual vascular network, but the shape of the formed networks still cannot be predicted and controlled, which limits the reproducibility of the model and its capacity to precisely mimic a specific existing vascular network. In another study, Agathe et al. were able to develop a new method of creating a self-assembled BBB *in vitro* model without using a microfluidic system. The model was made using a well plate containing a mixture of collagen microfibers and fibrin gel (Agathe et al., 2020). The mixture supported human ACs, PCs, and ECs, which eventually formed a 3D vascular network.

Furthermore, none of the aforementioned studies have assessed their model using TEER measurement, which is another major weakness that needs to be tackled in the self-assembly approaches.

### Effect of substrates on the cellular behaviors

The integrity of BBB culture models depends on several parameters. We have first investigated and analyzed existing 2D and 3D BBB models. These models use different substrates to support the cultured cells, directly influencing the functional cellular response. These substrates have the main role of mimicking the BM, which has a thickness ranging from 20 to 200 nm (Thomsen et al., 2017). Different substrate-related parameters were investigated and proved to have a great impact on cellular behavior, including nanoscale ordering (Dalby et al., 2007), substrates chemistry (Anderson et al., 2004; Nasir et al., 2021), substrate stiffness, and strain (Leipzig and Shoichet, 2009; Yeung et al., 2005), cell shapes, and substrate curvature (Baptista et al., 2019; Callens et al., 2020; Connon and Gouveia, 2021). Therefore, choosing an adequate substrate material and structure is fundamental to constructing the *in vitro* BBB model.

#### Substrates models

Depending on the material type and their fabrication method, three distinct substrates have been used in the construction of *in vitro* BBB models.

##### Porous membranes

Transwell and 2D microfluidic BBB models generally use synthetic semi-permeable porous membranes to support the cultured cells and realize the BBB’s permeability. The synthetic membranes are necessary components for *in vitro* BBB models, as they aid in the definition of the basal and laminal surfaces. Furthermore, these membranes have three primary functions: physically supporting the cultivated cells, compartmentalizing *in vitro* platforms, and ensuring the high-throughput ability. Different biocompatible materials have been applied to create the BBB models, including polycarbonate, polyethylene terephthalate (PET), PDMS, silicon nitride (SiN), and silicone dioxide (SiO_2_).

Early *in vitro* studies were limited to the utilization of ECM-coated PET and polycarbonate membranes with a thickness of 10 μm, which is fabricated using a polymer track-etching technique. The track-etching technique does not have a precise control; herein, the pores were randomly positioned with a diameter ranging from 0.4 μm to 8 μm (Booth and Kim, 2012). These two geometrical characteristics present a significant limitation for the recapitulation of the BBB integrity since they limit the cell-cell contact and thus paracrine and juxtacrine signaling (Kulczar et al., 2017). As a result, researchers used microfabrication techniques such as lithography to produce a new semi-permeable porous membrane known as an ultrathin membrane to address these restrictions. For instance, Carter et al. created ultrathin SiO_2_ membranes with either 0.1 μm or 0.3 μm of thickness with 3.0-μm micropores, which were patterned using photolithography at a density up to 22.7% (Carter et al., 2017).

**Figure 5:**
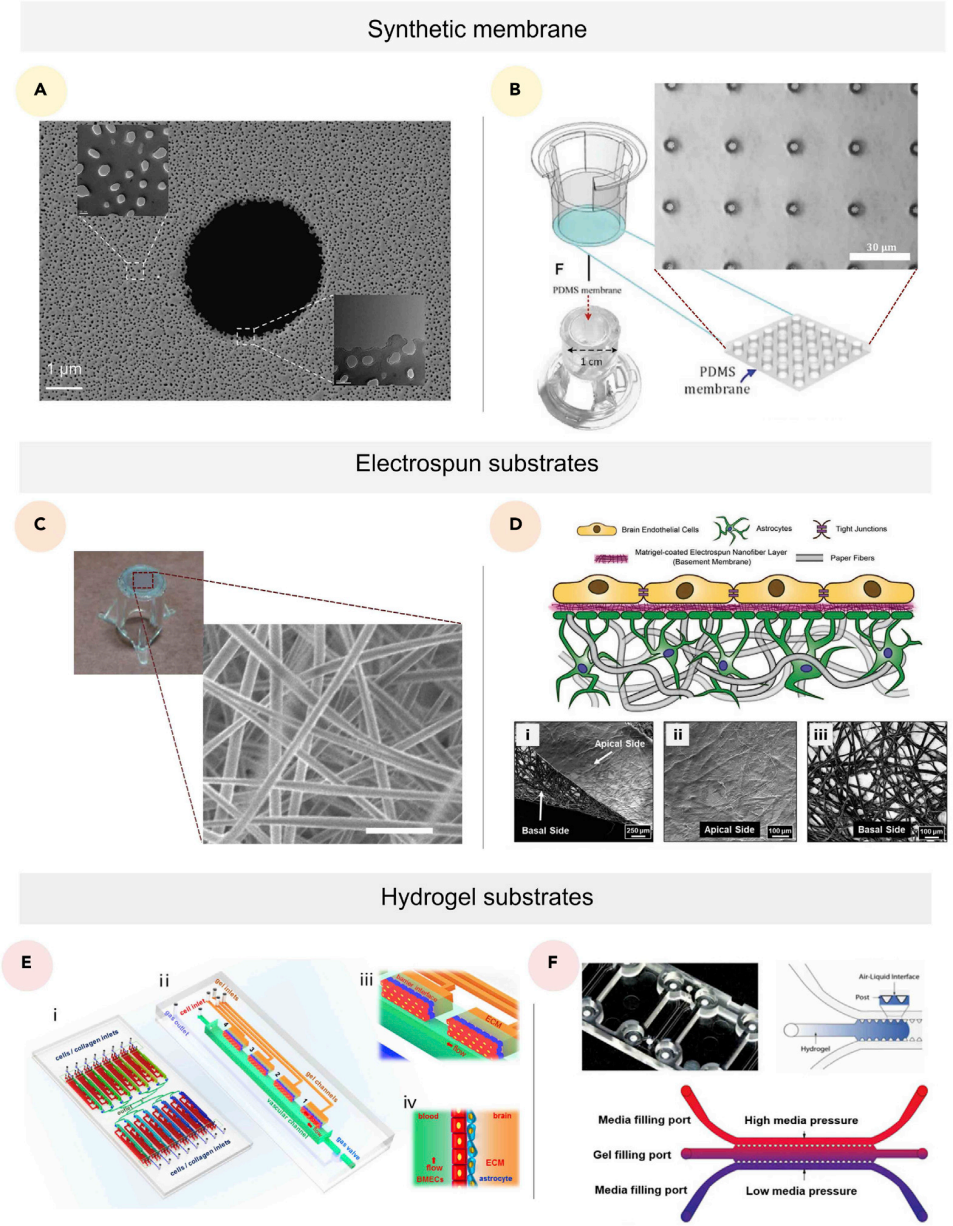
Substrate models for BBB (A) Scanning electron microscope image of dual-scale (nano- and microporous) synthetic membrane, with a scale bar of 1 μm (Salminen et al., 2019). (B) Schematic illustration (left) and bright-field image of the PDMS membrane, with a scale bar of 30 μm (right) (Zakharova et al., 2021). (C) Image of 15% electrospun and cross-linked gelatin biopaper attached to the bottom of the cell culture insert (left) SEM images of the electrospun fibers (right) with a sclae bar of 5 μm (Bischel et al., 2016). (D) Schematic of the 3D paper/nanofiber-based BBB model (top), and scanning electron micrograph of (1) the whole paper/nanofiber-based cell culture platform, with a scale bar of 250 μm; (2) the apical side of the platform, with a scale bar of 100 μm; (3) the basal side of the platform, with a scale bar of 100 μm (Huang et al., 2021). (E) Schematic illustration of the device design (1) composed of 16 units (2), containing two main channels separated by hydrogel substrates (3 and 4) (Xu et al., 2016). (F) Image and schematic illustration of a hydrogel substrate based BBB model (Buzhdygan et al., 2020).

However, SiO_2_ membranes cannot maintain cell positioning in the presence of shear stress, leading to detachment of the cultured cells (Mossu et al., 2019). Consequently, minimizing cell-substrate contact remains a valuable solution to increase the positional stability of the cells under dynamic shear stress, which can be realized by improving cell-cell contact. Therefore, there are two possible solutions, including the use of slightly larger pore diameters or increasing the density of the pores. Yet, previous studies showed that increasing pore diameter negatively impacts BBB integrity (Wuest et al., 2013). Thus, from this standpoint, it is vital to increase the density of the pores. Of interest, this interpretation was perceived by Salminen et al., which pushed the research team to create an ultrathin SiO_2_ membrane using the lithography microfabrication process with nanopores ranging from 15 to 100 nm in diameter and micropores with 3 μm in diameter (Figure 5A) (Salminen et al., 2019). As a result, the pore density was increased to 30%, which helped to improve the cells’ positional stability.

The membrane geometrical characteristics demonstrated to have crucial importance in the design of the *in vitro* models. These characteristics, however, are insufficient, and additional mechanical properties must be considered as well, as they have a significant impact on cellular behaviors (Bastounis et al., 2019; Yoo et al., 2020). The brain elastic modulus is less than 2.4 kPa whereas all the synthetic semi-permeable porous membranes besides PDMS membranes range from 1 MPa to 3 MPa. This tremendous difference between *in vivo* and *in vitro* mechanical environments was sufficient for Zakharov et al. to optimize PDMS membranes (a thickness of 2 μm), which can have an elastic modulus of around 3 MPa (Figure 5B) (Zakharova et al., 2021). Finally, besides having adequate mechanical properties, these silicone-based PDMS membranes are thinner and have better optical transparency and higher pore density.

##### Electrospun substrates

The electrospinning technology is a precise and versatile technology that allows the fabrication of a nanofiber-based platform. Several synthetic and natural polymers have been used to achieve electrospun membranes (Chen et al., 2021; Marino et al., 2021; Ozyurt et al., 2020; Patient et al., 2019). The fibrous structure produced using this method offers essential advantages that can ameliorate the functions of BBB models. Electrospun membranes have a comparably high surface/volume ratios, which means high pore densities. Therefore, these membranes can ameliorate cell-cell contact and facilitate their attachment to the membranes under dynamic conditions. Bischel et al. proposed a bilayer model to generate the *in vitro* BBB model (Figure 5C; Bischel et al., 2016). They created a customized gelatin “biopaper” electrospun membrane with a fiber diameter around 196 nm, an overall thickness of 4.5 μm, with an elastic modulus of 3.4 MPa. In recent work, Huang et al. adopted a hybrid nanofiber/paper substrate that used Matrigel-coated paper to support ACs and Matrigel-coated nanofibers from the other side to support brain ECs (Figure 5D; Huang et al., 2021). The thickness of the fibrous membrane can be controlled and varies between 0.2 and 5.5 μm. The hybrid approach was shown to have greater potential than the standard electrospun membrane since it provides a 3D scaffold that can support neuroglia growth and assures higher cell-cell contact. The controllability of the thickness of the nanofibrous membrane can also be used to create a cell-laden stratified layer to mimic the *in vivo* neuronal layers.

##### Hydrogel substrates

Self-assembled and 3D-patterned models usually both use hydrogels as substrates. Hydrogels can offer a convenient and structurally stable 3D microenvironment to the cultured cells. In fact, the brain is a very soft organ that has Young’s modulus less than 2.4 kPa (Budday et al., 2015). Therefore, the stiffness and viscoelasticity of hydrogels represent a major characteristic that favor their choice over other substrates to mimic soft tissues such as the brain. The tunability of hydrogels allows their use both as a scaffold containing neuroglia and supporting brain ECs or only as a thin membrane. Pellowe et al. employed a poly(ethylene glycol)-arginine-glycine-aspartic acid (PEG-RGD) hydrogel prepared by layering the hydrogel between two disposable zinc oxide layers and utilizing zinc oxide needles to build porous templates (Pellowe et al., 2017). The produced hydrogel membrane had a Young’s modulus of 96.78 kPa and a thickness of 18.89 μm, which is comparably thinner than previously reported PEG membranes but still thicker than *in vivo* ECM (Xu et al., 2019).

Besides synthetic hydrogels, natural hydrogels such as gelatin (Hsieh and Hsu, 2019), collagen (Diamantides et al., 2017), Matrigel (Berg et al., 2018), fibrin (Bayat et al., 2018), hyaluronic acids (Bonnesoeur et al., 2020; Ma et al., 2020a), and decellularized ECM (dECM) can offer a biomimetic microenvironment (Koh et al., 2018). In a work, Xu et al. created a unique PDMS microfluidic system that mimicked the *in vivo* brain ECM using rat tail high-concentration type-I collagen solution (Figure 5E; Xu et al., 2016). As a result, the device provided a high-throughput ability for examining brain tumor metastases and its therapy responses. Hydrogels are commonly utilized as a substrate in self-assembled models because their low stiffness has been shown to enhance the formation of endothelial vascular networks (Dessalles et al., 2021; Neuhaus, 2021; Ranadewa et al., 2021). For example, Li et al. injected fibrin hydrogel into a microfluidic channel to promote angiogenesis, allowing them to investigate BBB dysfunction caused by indoor nanoscale particulate matter (INPM) (Figure 5F; Buzhdygan et al., 2020).

### Cell sources

Besides chemical and mechanical cues applied by the substrates, dynamic conditions, and structure, cell source is another important parameter that influences the barrier integrity. Primary cells, immortalized cells, and stem cells have been frequently utilized to mimic the BBB *in vitro*. Primary cells offer low barrier permeability while maintaining BBB integrity. However, the requirement of animal sacrifice for each successive isolation remains a significant constraint and a time- and money-intensive process (Ahn et al., 2020; Buzhdygan et al., 2020; Lee et al., 2020; Shima et al., 2020). The use of primary porcine, bovine, and rodent cells is cheaper and easier to obtain compared to primary human cells. Multiple studies have shown that species differ in different aspects such as their morphology, their sensitivity to glutamate, and expression of ATP cassette binding (ABC) transporter family, which is important in determining BBB permeability (Oberheim et al., 2009; Takeuchi et al., 2006; Warren et al., 2009; Zhang et al., 2016a). As a result, while employing primary human cells is a prerequisite for approaching the human BBB, access to these cells is extremely limited. Immortalized cells are characterized by their reproducibility but could not achieve high TEER and low permeability values (Eilenberger et al., 2021; Jain et al., 2021; Kadry et al., 2021; Yu et al., 2020).

Current research focuses on replacing the aforementioned primary and immortalized cells with pluripotent stem cells that are self-renewable and have the ability to differentiate into various cell types in the body. For instance, Ge et al. cultured BMECs in contact with human embryonic stem cell-derived mesenchymal stem/stromal cells (hES-MSCs) in a 24-well format static Transwell model (Ge et al., 2019). They have used a pro-inflammatory cytokine (TNF-*α*) to induce the disruption of the BBB. As a result, hES-MSCs were able to repair the disruption and restore permeability and adhesion molecule expression. However, the use of embryonic stem cells raises ethical concerns. Contrarily, induced pluripotent stem cells (iPSCs) can be differentiated from adult somatic cells (Takahashi et al., 2007). Culturing hiPSC-derived cells is currently the most effective and precise approach to recreate the human BBB. Several protocols have been developed for the differentiation of each type of cells in the BBB. Lippman et al. were among the first to propose a protocol for differentiating iPSCs to brain ECs (Lippmann et al., 2012). This same protocol has been improved all along the years by the addition of retinoic acid during the differentiation process, which permitted the amelioration of the *in vitro* BBB functions to obtain a TEER value of around 5,350Ω cm^2^ under the co-culture condition (Lippmann et al., 2014).

A recent approach has been proposed by Neal et al. that suggests the replacement of a differentiation medium with a fully defined mixture of insulin, transferrin, and selenium (Neal et al., 2019). They achieved the current highest maximal TEER value for monoculture (∼8,000Ω cm^2^) and co-culture (∼10,500Ω cm^2^) models. Unlike iPSC-induced ECs, iPSC-induced PCs and ACs tend to take more time to differentiate. Interestingly, this was targeted by Aisenbrey et al. in a recent study, which reduced the differentiation time that was suggested in previous studies from 3 weeks to only 10 days (Neal et al., 2019; Zhang et al., 2017a). According to the protocol, on the sixth day of the differentiation, a CD34 magnetic-activated cell sorting (MACS) was used to separate the CD34^+^ endothelial progenitor cells from CD34^−^vascular cell population, which represents a key step in this process that accelerated the differentiation. The differentiation of ACs has also been studied to reduce the differentiation time by using CRISPR/Cas9-mediated inducible expression of NFIA or NFIA plus SOX9 human pluripotent stem cells (hPSCs) (Li et al., 2018). Consequently, the differentiation time was reduced from 3–6 months to 4–7 weeks. Neyrinck et al. proved that SOX9 overexpression using doxycycline in iPSC-derived neural progenitor cells was able to reduce the differentiation time to only 6 days (Neyrinck et al., 2021).

### Model assessment

To quantify the permeability of the *in vitro* BBB has been realized using various methods. Choosing the measurement methods can significantly impact the experiment findings, and thus this step should take as much attention as the previously described design parameters. Permeability assessment and TEER measurement are the most used methods (summarized in Table 2) since their combination allows the measurement of the transport of the charged and uncharged species across the BBB. Other assessing methods such as immunofluorescent staining of TJ proteins can complement permeability assay and TEER but are not sufficient to quantify the tightness of the BBB on their own (Hue et al., 2015; Zakharova et al., 2020).

**Table 2:** Overview of TEER and permeability measurements for different *in vitro* BBB models

Model	Cells	EC type	TEER	Permeability tracer	Permeability	References
Transwell	EC	hiPSC-derived BMECs	8,000 Ω cm^2^	Sodium fluorescein	10^−6^ cm/s	(Neal et al., 2019)
	EC, PC, NPC	hPSC	5,350 Ω cm^2^	Sucrose	5.7 × 10^−7^ cm/s	(Lippmann et al., 2014)
	EC, AC	hiPSC-derived BMECs	1,450 Ω cm^2^	Sucrose	3.4 × 10^−5^ cm/min	(Lippmann et al., 2012)
	EC, PC, AC	Primary rat cells	388 Ω cm^2^	NA	NA	(Nakagawa et al., 2007)
2D Dynamic	EC, PC, AC	hiPSC-derived BMECs	NA	Sucrose and Mannitol	4.923 × 10^−7^ cm/s and 6.760 × 10^−7^ cm/s	(Noorani et al., 2021)
	EC	hiPSC-derived BMECs	NA	Sucrose and Mannitol	1.883 × 10^−6^ cm/s and 2.380 × 10^−6^ cm/s	(Noorani et al., 2021)
	EC, PC, AC	hiPSC-derived BMECs	24,000 Ω	Fluorescent labeled of dextran tracers	9, 1.1, and 0.24 × 10^−8^ cm/s for 3,10, and 70 kDa	(Park et al., 2019)
	EC, PC	Immortalized mouse brain microvascular endothelial cells	260 Ω cm^2^	Mannitol	10^−6^ cm/s	(Wang et al., 2016)
	EC, PC, AC	Immortalized mouse brain microvascular endothelial cells	320 Ω cm^2^	Mannitol	0.5 × 10^−6^ cm/s	Wang et al., 2016)
3D Dynamic	EC, PC, AC	Primary human cells	NA	FITC-dextran	0.86 × 10^−6^ cm/s and 0.31 × 10^−6^ cm/s for 10 and 40 kDa	(Lee et al., 2020)
	EC, PC, AC	iPSC-EC	NA	FITC-dextran	2.2 × 10^−7^ cm/s and 8.9 × 10^−8^ cm/s for 10 and 40 kDa	(Campisi et al., 2018)
	EC, Rat cortical Neurons	human umbilical vein ECs	NA	FITC-dextran	0.45 and 0.36 × 10^−6^ cm/s for 20 and 70 kDa	(Bang et al., 2017)

Abbreviations: AC, Astrocyte; BMECs, Brain microvascular endothelial cells; EC, Endothelial cells; FITC, Fluorescein isothiocyanate; hiPSC, Human induced pluripotent stem cells; hPSC, Pluripotent stem cells; iPSC, Induced pluripotent stem cells; NPCs, Neural progenitor cells; PC, Pericyte.

#### TEER

**Figure 6:**
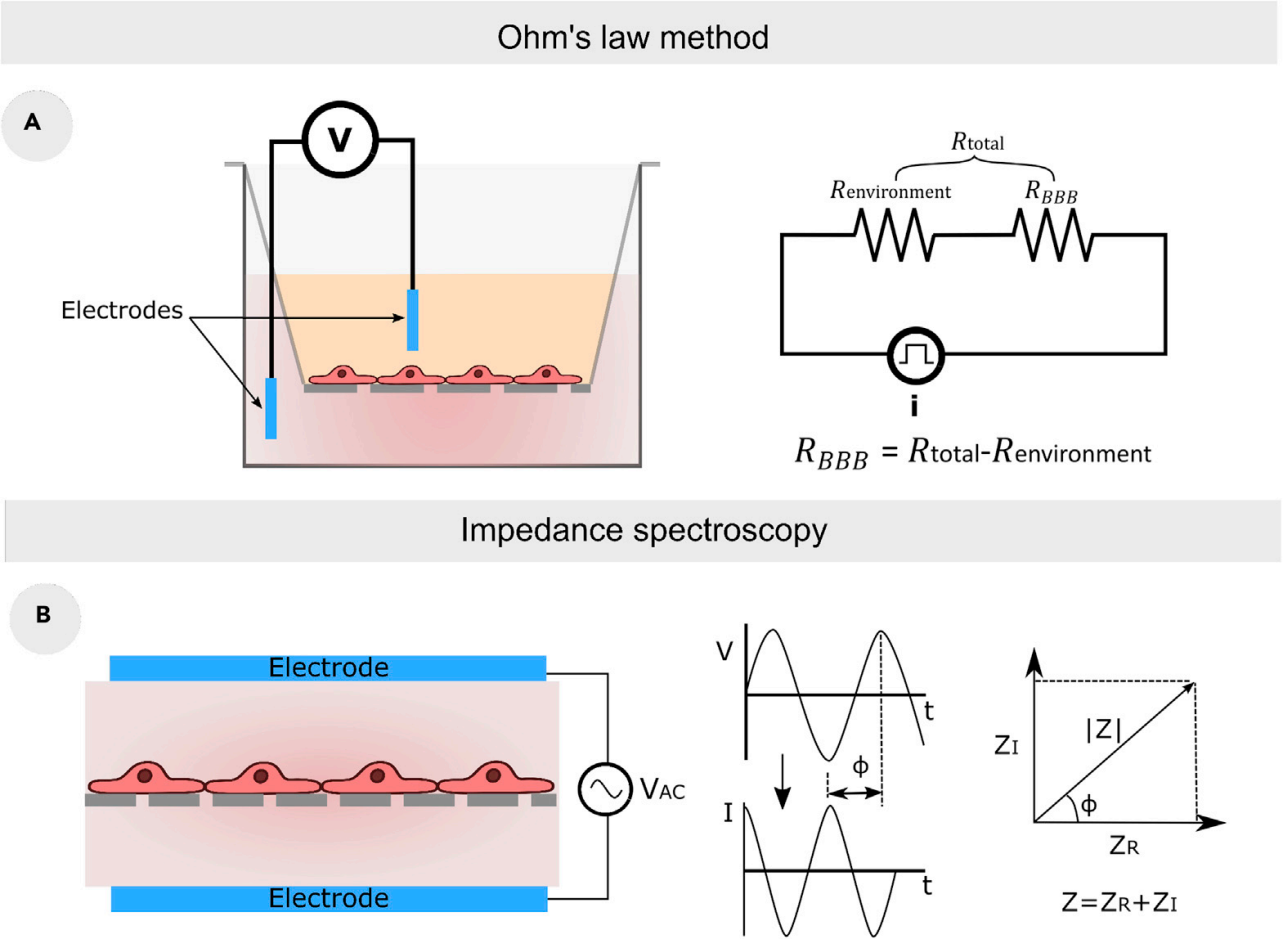
TEER measurement approaches (A) TEER measurement using Ohm’s law method with simplified equivalent circuit. (B) TEER measurement method based on impedance spectroscopy with the component of the impedance (Srinivasan and Kolli, 2019).

TEER measurement is a rapid and non-invasive quantitative method to assess the permeability of the BBB *in vitro* (Liang and Yoon, 2021; Srinivasan and Kolli, 2019). TEER translates the ionic resistance of the barrier in Ω·cm^2^ units. Therefore, the higher the TEER value, the tighter the barrier will be. Currently, there are two *in vitro* measuring methods: Ohm’s law method and impedance spectroscopy. Ohm’s law method is a direct and simple method to measure the resistivity of the barrier based on an equivalent electrical circuit of the *in vitro* model (Figure 6A). It mainly applies a direct (DC) or single frequency alternating current (AC) to measure the voltage and current in the whole model. As a result, the total resistance is obtained, and thus the resistance of the environment, which includes the resistance of the culture medium, electrodes, and the separating membrane, can be subtracted to finally obtain the BBB electrical resistance (*R*_*BBB*_).

The TEER value is usually expressed in Ω·cm^2^, and therefore the*R*_*BBB*_ is multiplied by the area of the culture membrane. The mispositioning of the electrodes and the heterogeneity of the electric field across the cell layer causes an obtaining of erroneous results. Therefore, while this method is widely utilized due to its simplistic setting, it is may not always be reliable. The impedance spectroscopy approach is based on sweeping the frequency of small amplitude to measure the amplitude and phase response of the resulting current (Figure 6B; Galla et al., 2011). As a result, researchers were able to quantify BBB tightness, cell proliferation, and cell differentiation using this technology (Benson et al., 2013; Galla et al., 2011; Gerasimenko et al., 2019).

The TEER measurement has been developed, and new concepts are being integrated. Henry et al., for example, constructed a PDMS microfluidic device with integrated electrodes, enabling for accurate real-time measuring and tracking of the influence of various cues on the microfluidic culture model (Henry et al., 2017). Tu et al. recently employed a similar approach which uses COMSOL simulation and micro-particle image velocimetry (μ-PIV) methods to develop a homemade electrode that also enables a more stable real-time measuring (Tu et al., 2020). However, these methods cannot measure the TEER value in specific parts of the model which may lead to inaccurate measurements (Odijk et al., 2015). Of interest, to override this limitation, Renous et al. proposed a new concept of TEER measurement called spatial-TEER which is based on the use of moving electrodes and consequently provide a localized TEER measurement in several points in the *in vitro* model (Renous et al., 2021).

#### Permeability

Besides the TEER measurement, the permeability assay has been widely used for static and dynamic models. Although the assay has the potential to impact cell structure, unlike TEER measurement, the intricacy of the model shape does not exclude its usage. The permeability assay is based on the measurement of the transport of molecules with different concentrations across the BBB. First, the total permeability *P* is calculated using the following equation (Yuan et al., 2010):

(Equation 3) $$P=\frac{\frac{\text{Δ}C_{A}}{\text{Δ}t}\times V_{A}}{C_{L}\times S}$$

with $\text{Δ}C_{A}/\text{Δ}t$ the variation of the marker concentration in the abluminal chamber by the difference of time, *V*_*A*_ the volume of the abluminal chamber, *C*_*L*_ the marker concentration in the luminal chamber, and S the surface area of the separating membrane. The obtained permeability is included both the permeability of the blank model (*P*_*blank*_) and the BBB (*P*_*BBB*_). Therefore, to obtain the permeability of the *P*_*BBB*_ in cm/s, the following equation is used (Yuan et al., 2010):

(Equation 4) $$P_{BBB}=\frac{P\times P_{\text{blank}}}{P_{\text{blank}}-P}$$

Several vascular permeability markers have been used, including fluorescently labeled FITC-dextran (Bischel et al., 2016; Marino et al., 2018; Salman et al., 2020), sodium fluorescein (Maeda et al., 2021; Xu et al., 2016; Zhang et al., 2021), lucifer yellow (Sitia et al., 2019), and the active permeability of P-glycoprotein (P-gp) substrate rhodamine 123 (Lippmann et al., 2014; Noorani et al., 2021).

## Recreating neuronal microenvironments for BBB *in vitro* models

### Neuron-BBB interactions

The critical role of ACs and PCs in BBB regulation has been widely explored and validated using *in vivo* and *in vitro* models. However, the neural niche comprises additional interesting cells such as microglia, oligodendrocyte precursor cells, and, most importantly, neurons. Previous *in vivo* studies demonstrated the contribution of microglia and oligodendrocytes that can be found elsewhere (da Fonseca et al., 2014; Haruwaka et al., 2019; Kimura et al., 2020; Wang et al., 2020; Watzlawik et al., 2010). When it comes to the influence that neurons may have on BBB, the investigation of neuronal-BBB interactions is hampered by the complicated structure and activities of the neural tissue.

Currently, only a few interaction mechanisms have been explored and elucidated (Andreone et al., 2015). Starting with the impact of neurons on the BBB, neurovascular coupling was the first mechanism that validated the presence of a functional interaction between neural cells and brain microvasculature where neural activation showed to increase blood flow (Presa et al., 2020; Zonta et al., 2003). Fascinating *in vivo* studies proved that vascular endothelial growth factor (VEGF)-A derived from neurons during early postnatal development regulates the CNS angiogenesis (Hogan et al., 2004; James et al., 2009; Ma and Huang, 2015). Additionally, Madelaine et al. were able to explore this interaction and found that miR-9 connects angiogenesis and neurogenesis by inhibiting the transcription factors TLX and Onecuts, which regulate VEGF-A expression (Madelaine et al., 2017).

Consequently, neurons influence the BBB formation, but their impact on the BBB permeability remains unclear. Recent advances in this field suggest that the excitation of neural activity using indirect optical or magnetic cues can affect the permeability of the BBB (Hrvatin et al., 2018; Vazana et al., 2016). Besides, abnormal high neural activities and the inhibition of some molecules, such as insulin-like growth factor 1 (IGF1), have been shown to increase the BBB permeability (Nishijima et al., 2010). Overall, the neural-BBB interaction proved to have a significant role in the BBB formation and, potentially, BBB permeability regulation which still needs to be further investigated.

The integration of the neurons in *in vitro* BBB models is a recent approach to mimic the brain microvasculature, which offers a more realistic microenvironment. The introduction of additional cell types, mostly neurons, into *in vitro* BBB models is thought to be a viable way to replicate the *in vivo* vascularized brain tissues. Two main approaches have been suggested in the literature, including the vascularization of brain organoids and the culture of neurons in *in vitro* BBB models to form a microfluidic or Transwell NVU model.

### NVU models

#### NVU Transwell models

The key characteristics of NVU Transwell models are the same as those of BBB Transwell models. They are indeed considered the simplest models in terms of reproducibility and architecture. Schiera et al. were among the first to attempt to develop an NVU Transwell model by co-culturing RBE4.B immortalized rat BMECs, cortical neurons, and ACs (Schiera et al., 2003, 2005). The researchers compared NVU and BBB models using sucrose permeation assays, which confirmed the importance of integrating neurons alongside other neuroglia to reduce BMECs layer permeability. However, the model still has two obvious limitations, which are their use of rat BMECs out of human BMECs and the absence of PCs. A recent NVU Transwell model, constructed by Stone et al., co-cultured four primary human cells, including HBMECs, ACs, PCs, and neurons, which were used to validate the important role that neurons play in response to ischemia and their participation in the upgrade of the BBB sensitivity to oxygen-glucose deprivation (Stone et al., 2019).

#### NVU microfluidic models

**Figure 7:**
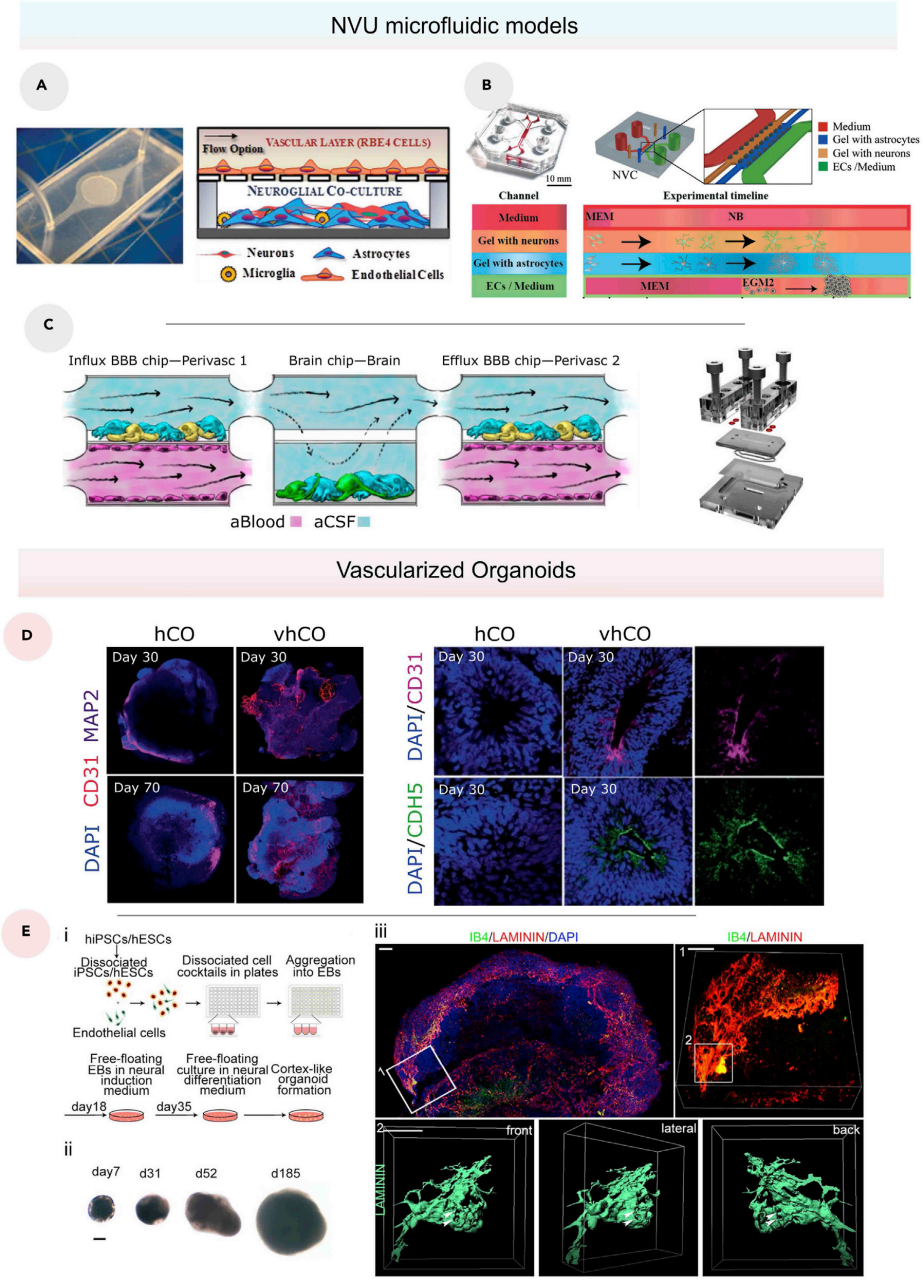
NVU models (A) Photo (left) and schematic Illustration (right) of the setting of the neurovascular co-culture model (Achyuta et al., 2013). (B) Photo of the neurovascular model (left), schematic layout of the neurovascular model containing four channels: two central hydrogel regions for co-culturing ACs (blue) and neurons (orange) and two side channels containing ECs (green) and media (red) (Adriani et al., 2017). (C) Schematic of a coupled BBBoC model containing hBMVECs (magenta) on all four walls of the lower vascular compartment and a mixture of brain ACs (blue) and PCs (yellow) in the top compartment of both BBB chips, and human brain neuronal cells (green) and ACs (blue) in the lower compartment of the brain chip (Maoz et al., 2018). (D) Immunostaining of whole-mount vascularized human cortical organoids (vhCOs) and control human cortical organoids (hCOs) at different time points (days 30 and 70) for CD31 and MAP2 (left), and immunostaining for CD31 and CDH5 in sectioned hCOs and vhCOs at day 30 (right) (Cakir et al., 2019). (E) Schematic diagram of the biofabrication process of the vascularized cerebral organoid (1), Representative bright-field images of vascularized organoid with a scale bar of 200 μm (2), Whole mount imaging of the vascularized organoids on day 42 (3) (Shi et al., 2020).

The microfluidic technology presents an efficient tool to create a dynamic microenvironment for engineered tissues (Mehta and Rath, 2021; Zhang and Khademhosseini, 2020). Therefore, the tendency toward NVU-on-a-chip models instead of Transwell models is a natural consequence of recent technology development. Achyuta et al. created a PDMS microfluidic device with two chambers separated by a polycarbonate porous membrane, one of which cultures rat brain endothelial cell line (RBE4) under dynamic flow, and the other cultures including neurons, ACs, and microglia at the same time (Figure 7A) (Achyuta et al., 2013). Brown et al. used primary human BMECs, PCs, ACs, and hiPSC-derived human cortical neurons to propose a more precise technique employing a similar two-chamber approach (Brown et al., 2016). The devices enabled the researchers to study the BBB’s reaction to lipopolysaccharide and a cytokine cocktail consisting of interleukin-1 (IL-1), tumor necrosis factor alpha (TNF-*α*), and monocyte chemoattractant protein-1/2 (MCP-1/2). Adriani et al. developed a more modern strategy in which neurons and ACs are separated into two parallel channels attempting to mimic the *in vivo* structure (Figure 7B; Adriani et al., 2017). Therefore, the model comprises three channels supporting each cell type separately, with an additional channel containing a minimum essential medium (MEM).

From another point of view, instead of using one organ-on-a-chip model, Maoz et al. considered connecting three different chips to investigate the new metabolic coupling between neural tissue and BBB (Figure 7C; Maoz et al., 2018). In most cases, coupling multiple organs-on-chips (OOCs) is accomplished by connecting separate OOCs that imitate distinct organs; however, the proposed device by the same research team demonstrated a unique approach of coupling multiple OOCs that mimic the same organ but have diverse functions. Their first chip is a BBBoC that simulates influx across the BBB, followed by a brain tissue model and a BBBoC that simulates efflux across the BBB.

Angiogenesis models were also used to create NVU-on-a-chip models. From the same approach of angiogenesis-based BBBoC models, NVU models were constructed using microfluidic devices mainly composed of three compartments separated by micropillars where the central compartment usually contains a self-assembled vascular network, and the other two compartments support neurons and ACs (Bang et al., 2017). According to Uwamori et al., the angiogenesis-based model can be improved further by injecting fibrin-Matrigel into the central compartment, which can promote not only angiogenesis but also neurogenesis (Uwamori et al., 2017).

#### Vascularized organoids

Brain organoid models gained great interest recently. Generally, they are produced from stem cells or organ progenitors to self-organized spheroids capable of mimicking complex organ functions (Clevers, 2016; Heydari et al., 2021; Nishikawa et al., 2020; Rozich et al., 2020). Brain organoids, specifically, have numerous potentials for recreating accurate 3D neural structures and simulating neurogenesis, neuronal migration, neuronal maturation, and neural connections (Agarwal et al., 2021; Shou et al., 2020; Thakor et al., 2020). Vascularized brain organoids have been targeted for primarily one reason, which is supplying the organoids with oxygen and nutrients (Rawal et al., 2021). However, the current findings hold a promising prospect for future NVU organoid models. For instance, Mansour et al. transplanted hPSC-derived brain organoids into the retrosplenial cortex of non-obese diabetic/severe combined immunodeficiency (NOD-SCID) mice, and the organoids survived for 233 days post-implantation (Mansour et al., 2018). During the implantation, the model demonstrated axonal extension and developmental dynamics, including neural progenitor cells (NPCs) maturation and differentiation, synaptogenesis, and gliogenesis, which were consistent with previous findings (Camp et al., 2015; Lancaster and Knoblich, 2014). Of interest, the organoid showed a robust integration in the host brain with an extension of the host’s vascular system in the graft. The study also found evidence of functional synaptic connectivity between the host and the graft, albeit this needs to be investigated further.

The vascularized organoids’ ability to connect with *in vivo* tissues is an important advantage that other research approaches could not achieve. Pham et al. presented an organoid coating with Matrigel, which promoted robust vascularization (Pham et al., 2018). However, such organoids did not last more than 2 weeks *in vivo*. Considering the importance that BBB has in neural tissues, these two previous applications cannot be considered as NVU models since they do not exhibit any specialized BBB functions. Interestingly, this limitation has recently been addressed by Cakir et al. (Figure 7D; Cakir et al., 2019). They created vascularized human cortical organoids derived from human embryonic stem cells. They employed ETV2-induced EC-reprogramming in brain organoids to generate a BBB-like structure with increased expression of TJ proteins such as ZO-1 and occludin.

In another study, Shi et al. vascularized human brain organoids using HUVECs (Figure 7E; Shi et al., 2020). The research study concluded that the co-culture of the organoids and HUVECs induces the expression of P-gp, which indicates that the HUVECs adopted a brain-like EC fate. Overall, current vascularized organoid models have great potential in mimicking the 3D microenvironments of the brain but fail to replicate the BBB functions, yet the technology is still in its early development steps toward accurate modeling of the NVU.

Overall, the advantages and disadvantages of the NVU Transwell and microfluidic models are the same as those of the BBB Transwell and microfluidic models. The implementation of additional neural cells allows the investigation of possible interactions between the BBB and neuroglia. The dynamic conditions of microfluidic models offer the control of chemical and mechanical parameters. The ability to quickly extract relevant information from Transwell and microfluidic NVU models utilizing sensors like TEER is also a significant advantage limited in vascularized organoid models. The simplicity of Transwell and microfluidic models is a key in replicating the model, which helps assess the consistency of the obtained results and thus improves its reliability. Contrarily, vascularized brain organoids take a longer time to construct and have comparably lower accessibility which hinders the model’s efficiency. However, the organoids’ 3D structure aids in better reproducing brain cytoarchitecture and cell-cell interactions, allowing for functional synaptic connectivity, which has yet to be realized using other *in vitro* models.

## Challenge and future perspectives

Current advances in BBB *in vitro* models rely on a plethora of advanced techniques, which have helped to improve the approach of the structural and cellular complexity of the *in vivo* BBB. The facile reproducibility of Transwell models allowed the investigation of the efficiency of the different mono/co-culture settings. However, these models present a significant caveat that limits the recreation of the *in vivo* dynamic microenvironments. The synergy of microfluidics and *in vitro* culture produces a more efficient tool to better mimic the dynamicity of the *in vivo* tissues, more precisely the BBB and NVU. However, the construction of 3D hollowed network structures of *in vitro* BBB models has not been achieved. A similar limitation representing a significant challenge in the biofabrication of normal vasculature has been addressed using sophisticated 3D printing and bioprinting methods, including inkjet, laser-assisted, micro-extrusion, and vat-polymerization bioprinting (Aazmi et al., 2021; Santoni et al., 2021; Wang et al., 2021; Zhu et al., 2021b).

Furthermore, although neural tissues have also been successfully bioprinted, they have yet to be vascularized (Knowlton et al., 2018; Rouleau et al., 2021). The CNS cells’ heterogeneity and proximity eventually hinder the application of these methods to fabricate an accurate 3D BBB model. The distance between ACs and BBB ECs or PCs ranges from ∼20 nm to ∼100 nm, whereas stereolithography/digital light processing bioprinting, which has the highest resolution among common bioprinting methods, can only achieve a printing resolution of ∼50 μm when producing cell-laden constructs (Miri et al., 2019). Therefore, direct bioprinting methods may not be able to respond to the presented need. However, sacrificial bioprinting, an indirect bioprinting method, has shown to be the most adequate to construct *in vitro* lumenized vasculature (Duchamp et al., 2019; Li et al., 2020; Shao et al., 2020; Zhang et al., 2016b, 2017b). This method is based on using a fugitive material that will be removed by external cues such the temperature. The supporting material remains, and cells are injected into the hollowed structure to generate a thin layer of ECs. Potentially, to recreate the NVU, the supporting material can support a mixture of neurons, ACs, and other neuroglia. After removing the sacrificial material brain, ECs can be seeded to form the BBB in the hollowed structure. As a result, utilizing this indirect method, the requirement of cell proximity to assure cell-cell interactions can be possibly fulfilled without resorting to high-resolution biofabrication methods.

Besides the need to recreate complex 3D structures, *in vitro* substrates have revealed very different mechanical properties compared to the *in vivo* substrates. In fact, lowering the stiffness and strain of the substrates will directly affect the integrity of the model, and thus it will be more challenging to maintain a hollow structure. Herein, this mechanical properties tradeoff represents a dilemma that is commonly addressed using composite materials such as cellular and porous composite materials since the stiffness decreases with the square of porosity (Sadasivuni et al., 2018). Yet, porous materials cannot mimic the low permeability that BM and GCX have; herein, stratified composite materials composed of different layers, including porous materials and dense polymers, have a more significant potential to mimic the mechanical properties of the neural tissues. Moreover, existing *in vitro* models have overlooked the critical significance of GCX, which we mentioned previously, and have thus neglected to include it in their models. Therefore, a luminal coating of the BBB using a high-density polymer that replicates GCX should be considered in future research works.

Cell ratios in the CNS differ from one region to another to perform different functions and are considered an important parameter to define and understand various aspects of the brain (von Bartheld et al., 2016). However, only a few research works have addressed attention to this crucial parameter (Achyuta et al., 2013; Brown et al., 2019b; Lee et al., 2020). Cell ratio quantification is a tool that can be used either during the design process of the *in vitro* model or as an assessment measurement to verify whether it approaches the *in vivo* ratios. Yet, angiogenic models show a variable ratio that changes by time based on several complex parameters, and thus makes the ratio hard to preserve. Recent studies utilized machine learning (ML) as a powerful tool that can predict the development of different cells (Ing et al., 2017; Sadasivuni et al., 2018; Strobel et al., 2021). As a result, using ML to create more accurate angiogenic models, cell ratios, and sprouting shapes can eventually be predictable. Besides, we noted the absence of different neuroglia such as oligodendrocytes and NG2-expressing cells in current BBB models, although they clearly contribute to the maintenance of the BBB.

The advancement of the BBB models toward an accurate model, which is the NVU, has been achieved by co-culturing neurons with BBB cells. Yet, it is essential to note that the composition of the neural tissue depends on its location in the brain. This is also valid for smaller-scale regions, for example, in the cerebellum, where the six layers of the neocortex or the layers of the allocortex are shown to have different cell types and structures (Posimo et al., 2013; Schmid et al., 2019). From a biomechanical standpoint, these regions can also have different mechanical properties, as the white and gray matter of the brain did (Bilston, 2011; Budday et al., 2015). Therefore, the replication of the *in vivo* NVU should be precisely targeted by carefully selecting cell types that are present in that same region in the brain with precise ratios.

The NVU models represent a step toward the brain-on-a-chip. Besides the neural tissue and the vasculature, the brain contains the interstitial system (ISS). The ISS is composed of mesenchymal fluid (ISF) and ECM, occupying 15–30% of the brain volume (Lei et al., 2017). Therefore, future amelioration must consider the integration of the ISS in the NVU models. In fact, coupled organs on chips have the potential to recreate interacting tissues from the same organ or different organs, which will play an important role in the replication of various brain functions.

Finally, assessing the BBB and NVU models is a very delicate step. The TEER measurement is usually hampered by the structural complexity of *in vitro* models, leading to erroneous results. The *in vivo* and *in vitro* assessing methods are still incoherent and do not allow accurate comparisons and thus validation. Besides, several recent *in vivo* studies used transcriptomic analysis to assess the BBB (Munji et al., 2019; Sabbagh et al., 2018; Vanlandewijck et al., 2018; Zhang et al., 2014). As a result, analyzing transcriptome profiles of NVU cells is now regarded a critical step in evaluating and improving *in vitro* models.

## Conclusion

The significance of generating accurate BBB *in vitro* models has driven researchers to incorporate more advanced technologies into their biofabrication processes. First, by producing thinner and more suitable membranes, significant improvements in static models have been accomplished. The use of microfluidic technology to create dynamic circumstances has improved these models even further, allowing them to build precisely controlled BBBoCs with more realistic forms. From another perspective, the complexity of the real BBB has been approached by introducing more cell types to create three main settings, including monoculture, co-culture, and tri-culture. The neuronal microenvironment has a big impact on the BBB. Therefore, a synergic approach was developed based on the integration of neurons and additional neuroglia in BBB models either by using standard microfluidic systems or vascularizing neural organoids. However, the potential of NVU models and new bioprinting techniques is still unexplored but remains promising to create better vascularized neural tissues.
